# Does childhood health assessment questionnaire can predict outcome of patients with juvenile idiopathic arthritis?

**DOI:** 10.1186/1546-0096-9-S1-P160

**Published:** 2011-09-14

**Authors:** G Susic, R Stojanovic, I Soldatovic, N Damjanov, G Radunovic

**Affiliations:** 1 Institute of Rheumatology Belgrade, Serbia; 2 Medical Statistics, School of Medicine, University of Belgrade, Serbia

## Aim

To assess the ability of Childhood Health Assessment Questionnaire (CHAQ) in predicting outcome of patients with juvenile idiopathic arthritis (JIA).

## Method

87 pts. (f 69%, m 31%) average age 14,1 yrs. disease duration 5,2 yrs., were follow up for 3,7 (2-5) yrs. Parents/patients over 12 yrs. completed CHAQ at the beginning and at the end of study and disability index (DI) was calculated. CHAQ DI=0 was considered as normal, 0,125-0,5=mild, ≥0,6=moderate/severe disability. Fifty nine (67,8%)pts. were treated with methotrexate, 42 (52,8%)pts. with etanercept. Outcome was defined as active disease or remission (Wallace criteria) [1]. We used regression models for the assessment predictive strength of CHAQ.

## Results

CHAQ DI at the baseline was 0,541, at the end of follow up 0,398 (p<0,05). Number of patients with moderate/severe disability decreased from 29 to 18 (33,3% vs. 20,7%, p<0,01). Number of patients in remission increased from 15 to 47 (17,2% vs.54,0%, p<0,001). Seventy (80,6%)pts. with normal CHAQ DI at baseline, were in remission at the end of study, while 66 (75,9%)pts. with moderate/severe CHAQ DI at the beginning had active disease at the last visit. CHAQ showed good predictive ability for the disease outcome (71,3%). Odds ratio for having active disease for patients with mild CHAQ DI was high (OR 3,33, CI=1,034-10,746, p=0,044), for patients with moderate/severe CHAQ DI was very high (OR 13,095, CI=3,821-44,882, p<0,001), compared to patients with normal CHAQ value. Patients with moderate/severe CHAQ DI had hazard ratio for the active disease 4,959 (CI=1,855-11,385, p<0,001).

## Conclusion

CHAQ demonstrated good ability in predicting outcome of the disease. Due to its simplicity and accessibility it is useful in everyday clinical practice for identifying patients with poor prognosis.
